# Gas Chromatography–Mass Spectrometry (GC-MS) in the Plant Metabolomics Toolbox: GC-MS in Multi-Platform Metabolomics and Integrated Multi-Omics Research

**DOI:** 10.3390/ijms27031343

**Published:** 2026-01-29

**Authors:** Nadezhda Frolova, Tatiana Bilova, Svetlana Silinskaia, Anastasia Orlova, Anastasia Gurina, Andrej Frolov

**Affiliations:** 1 Laboratory of Analytical Biochemistry and Biotechnology, K.A. Timiryazev Institute of Plant Physiology, Russian Academy of Sciences, 127276 Moscow, Russia; frolovanadja@yandex.ru (N.F.); svetlanasilsv@mail.ru (S.S.); lanas_95@mail.ru (A.O.); anastasia.gurina@list.ru (A.G.); 2 Department of Plant Physiology and Biochemistry, St. Petersburg State University, 199034 St. Petersburg, Russia; 3 Institute of Basic Biological Problems, Pushchino Center for Biological Research, Russian Academy of Sciences, 142290 Pushchino, Russia; 4 Higher School of Living Systems, Institute of Medicine and Life Sciences, I. Kant Baltic Federal University, 236041 Kaliningrad, Russia

**Keywords:** GC-MS, metabolite profiling, metabolomics platforms, multi-omics, plant metabolomics, primary metabolites

## Abstract

Innovative developments of GC-MS over the last two decades made this methodology a powerful tool for profiling a broad range of volatile metabolites and non-volatile ones of non-polar, semi-polar and even polar nature after appropriate derivatization. Indeed, the high potential of GC-MS in the analysis of low molecular weight metabolites involved in essential cellular functions (energy production, metabolic adjustment, signaling) made it the method of choice for the life and plant scientists. However, despite these advances, due to their intrinsic thermal lability, multiple classes of hydrophilic low-molecule weight metabolites (like nucleotides, sugar phosphates, cofactors, CoA esters) are unsuitable under the high-temperature conditions of the split–splitless (SSL) injection and GC separation, which makes the analysis of such compounds by GC-MS challenging. Therefore, to ensure comprehensive coverage of the plant metabolome, the GC-MS-based metabolomics platform needs to be efficiently combined with other metabolomics techniques and instrumental strategies. Moreover, to get a deeper insight into dynamics of plant cell metabolism in response to endogenic and exogenic clues, integration of the metabolomics data with the output obtained from other post-genomics techniques is desired. Therefore, here, we overview different strategies for the integration of the GC-MS-based metabolite profiling output with the data, acquired by other metabolomics techniques in terms of the multi-platform metabolomics approach. Further, we comprehensively discuss the implementation of the GC-MS-based metabolomics in multi-omics strategies and the data integration strategies behind this. This approach is the promising strategy, as it gives deep and multi-level insight into physiological processes in plants in the systems biology context, with consideration of all levels of gene expression. However, multiple challenges may arise in the way of integrating data from different omics technologies, which are comprehensively discussed in this review.

## 1. Introduction

**Figure 1:**
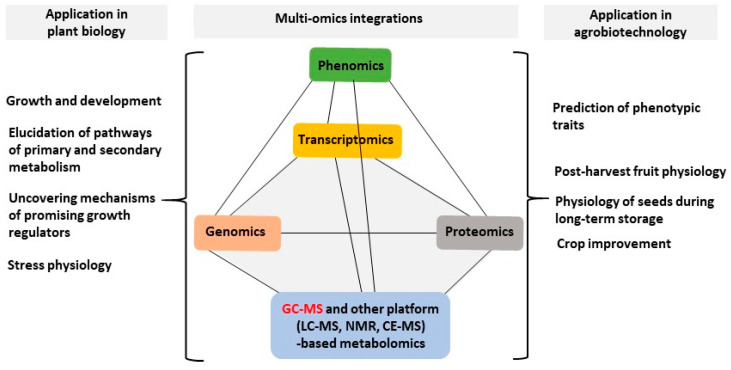
Multiple omics experiment with implementation of GC-MS analytical platform and its potential applications in plant biology and agrobiotechnology sciences. Color nodes indicate major omics platforms, while the integrations between them are indicated with edges.

Due to the rapid development of new post-genomic methodological platforms—transcriptomics, proteomics, metabolomics and phenomics—valuable information about the corresponding global sets of gene products, i.e., transcriptome, proteome and metabolome, as well as their dynamics in response to external and internal clues, can be accessed. This information is essential for understanding how an organism’s characteristic traits (phenotype) form. Obviously, a well-coordinated parallel investigation of these sets might give new insights into gene functions and mechanisms behind the organism’s fine regulation of metabolism, stress response and development. Indeed, genomics targets understanding the genetic basis of phenotypes; transcriptomics targets the changes in transcriptome, i.e., the population of mRNA molecules responsive to the internal or external stimuli; proteomics addresses the corresponding changes in protein dynamics; whereas metabolomics delivers valuable information about the resulted shifts in cellular metabolism at the level of small effector molecules [1,2,3,4]. Lastly, phenomics analyzes the properties of a phenotype and the processes of its formation during ontogenesis and in response to environmental factors. Therefore, integration of these information layers allows the implementation of the systems biology tools and delivers an integrative overview of the molecular regulation within the cell and in the whole organism [5,6] (Figure 1). This strategy has found essential application in multiple areas of plant biology and agrobiotechnology.

In solving the actual problems of the systems biology, the state-of-the-art metabolomics serves as a vital information source offering metabolic solutions and giving access to fine regulatory mechanisms behind cellular and organism responses [5,7]. In terms of the metabolomics approach, dynamics of individual metabolites—the ultimate products of gene expression—can be characterized. Therefore, a comprehensive phenotypic assessment of a biological system can be achieved. On the other hand, metabolites can act as the substrates, intermediates and products of enzymatic and non-enzymatic reactions occurring in the organism. They are not directly encoded in the genome, and their biosynthesis often involves multiple enzymes. Therefore, some metabolites might be stoichiometrically interconnected. This and other features specific for metabolites underlie the complex metabolic networks, which are not characteristic for proteome and transcriptome [8,9]. Thus, metabolomics experiments might deliver valuable functional information that is crucial in systems biology research.

From a methodological point of view, metabolomics offers a combination of various approaches, each of which allows the simultaneous analysis of only a certain group of metabolites—the method usually referred to as metabolite profiling [10,11]. Due to impressive diversity of the plant metabolome, individual metabolite classes are too different in their physicochemical properties to be efficiently analyzed by only one metabolomics technique. Therefore, several metabolomics platforms need to be combined to gain the best possible metabolome coverage and to obtain the most comprehensive information on the plant metabolite network and related regulatory pathways [12,13]. This strategy often serves as the optimal solution to address the changes in metabolome accompanying development and stress responses [4,14].

Among the available analytical platforms, which are currently employed in the state-of-the-art metabolomics workflows, gas chromatography coupled online to mass spectrometry (GC-MS) still remains one of the most widely utilized techniques. Indeed, in combination with appropriate derivatization strategies, GC-MS is ideally suited for analysis of diverse low molecular weight plant metabolites with essentially different properties, i.e., not only volatiles, but also non-volatile compounds of non-polar and even polar nature can be analyzed by this technique [10,15,16]. Importantly, the processing of the acquired GC-MS data relies on the combination of unique retention indices (calculated based on the retention time (t_R_) calibration) and compound-specific electron ionization (EI)-MS spectra acquired at the standardized energy of 70 eV. This combination makes the whole GC-MS setup extremely robust and reliable [17]. In addition, GC-MS instrumentation is typically relatively inexpensive and is easy in operation. However, despite these obvious advantages, adequate analysis by GC-MS assumes relatively low molecular weight, high thermal stability and volatility of the analytes or/and their derivatives. In the context of these considerations, some classes of primary metabolites (e.g., nucleotides, carbohydrate-phosphates, cofactors), as well as secondary metabolites (e.g., alkaloids, polyphenol conjugates and polycyclic terpenes), are incompatible with efficient and reliable GC-MS analysis [18,19]. Therefore, to get access to the most representative sets of primary and secondary metabolites involved in central energy and various biosynthetic pathways, it is necessary to complement the GC-MS methodology with one or several orthogonal analytical platforms, which would cover the metabolite classes hardly accessible by GC-MS. Further integration of the outputs from different metabolomics platforms at the data interpretation step would enable a comprehensive characterization of the target plant physiological responses at the molecular level [2,4,20]. This approach, particularly when integrated with other omics techniques, is rapidly becoming the central focus of metabolomics research [21]. 

Therefore, here we comprehensively review the existing multi-platform metabolomics strategies over the past decade focusing on the integration of the GC-MS-based workflow with other metabolomics platforms and discuss the prospects of its implementation in multi-omics concepts. 

## 2. Integration of the GC-MS-Based Workflow with Other Metabolomics Platforms

### 2.1. Strengths of Multi-Platform Metabolite Profiling

As it was mentioned above, the chemical diversity of the metabolome and its significant size pose serious challenges for analytical profiling; that is why only a part of the total plant metabolome can be addressed with one specific instrumental platform. Therefore, the metabolomics datasets, typically acquired in terms of the currently existing workflows, represent only subsets of complex metabolic profiles [22]. It is worth noting that each of analytical methods used in metabolomics is featured with characteristic resolution and sensitivity, which are adjusted with consideration of the chemical and physical properties of the target analyte groups. Nevertheless, the dynamics of metabolite patterns, associated with development- or stress-induced physiological shifts is a kind of puzzle in which it is necessary to consider as many individual metabolic changes as possible [23,24,25]. In this context, obviously, the overall success of the metabolomics analysis to a high extent is defined by the number of metabolites addressed. Therefore, using a combination of different instrumental platforms might increase the numbers of identified individual compounds, i.e., might improve metabolome coverage and the overall analytical efficiency [26]. Indeed, over the recent decade, the parallel implementation of different instrumental platforms became a gold standard in the metabolomics research. Most often, the integrative strategy also allows the scheduling of sample preparation procedures for all individual techniques in a general and universal multi-staged workflow, including specific sequential steps to prepare samples for GC–MS, liquid chromatography–mass spectrometry (LC–MS) and/or spectroscopic analyses [27,28]. Typically, it appears to be possible to organize such methods as multi-well high-throughput protocols, which are especially suitable for large-scale experiments. Furthermore, the outcomes of corresponding analytical techniques complement each other or even partly “overlap” by their metabolome coverage, which appears to be advantageous [28,29]. Indeed, it provides an opportunity to compare (via cross-validation approach) the potential of each method within the integrated setups for the analysis of specific individual metabolites (both in the identification and quantification aspects) [30,31,32]. 

Therefore, when designing a multi-platform metabolomics strategy, selection of the techniques to be combined with GC-MS is the key question. In this context, some general and GC-specific considerations need to be addressed.

### 2.2. Cross-Validation in Multi-Platform Metabolite Profiling

Generally, when combining different methods, their target analyte groups need to be well understood and well defined. For this, in each case, validation experiments might be useful for selecting the most appropriate analytical approach and for precise optimization of the selected method. In terms of such validation, Koistinen and colleagues [33] compared the metabolome coverage obtained with different analytical platforms, including several LC-quadrupole-time of flight (QqTOF), one LC-quadrupole ion trap (QTRAP), one LC-quadrupole (Q)-MS, and two GC-MS instruments. The authors proposed an analytical coverage quality control mix consisting of twelve chemical standards, which covered a broad chemical spectrum representing different groups of natural plant products. Based on such information, a specific strategy for integration of LC- and GC-MS methodologies can be developed. 

To date, the cross-validation approach is best-established for the analysis of amino acids and their derivatives, which can be accessed not only by GC-MS or GC–flame ionization detection (FID), but also by reversed-phase (RP)–ion-pair reversed-phase (IP-RP), hydrophilic interaction chromatography (HILIC)–(ultra) high performance liquid chromatography (U)HPLC-MS or capillary electrophoresis (CE)-MS, fluorescence or ultraviolet-visible spectroscopy (UV–vis) detection [34]. The combination of those techniques for the comprehensive profiling of amino acids was convincingly proven to increase the overall efficiency and reliability of the analysis [35,36]. Recently, this approach was successfully extended to other metabolite classes. For example, Hazrati and colleagues employed LC-QqQ-MS, LC-QqTOF-MS and GC-TOF-MS in a parallel global metabolite profiling of benzoxazinoids in rye [28]. After analysis with univariate and multivariate statistics, the results of such experiments allowed selection of the most suitable methodology to characterize the aglycones and glycosides of benzoxazinoids. This finding was in agreement with the results of other studies, which indicated that multi-platform metabolomics yields the most complete and representative metabolite profiles of the plant objects on study [37,38]. 

### 2.3. Analytical Potential and Limitations of GC-MS

Considering specifically the analytical potential of GC-MS, it should be noted that the application area of this method is limited to exclusively volatile analytes [39]. Usually, volatile nonpolar (e.g., terpenes, fatty acid esters) and even small polar organic compounds (e.g., low-molecular-weight aliphatic and aromatic alcohols) are the proper analytes for GC-MS. However, the non-volatiles represented by hydrophilic compounds of higher or lower molecular weight, need to be chemically modified to obtain their volatile and thermally stabile derivatives prior to GC-MS analysis [40]. One of the most widely employed derivatization strategy is trimethylsilylation (TMS), which can be efficiently applied to the most of the low molecular weight thermally stable polar primary metabolites (amino acids; amines and polyamines; carboxylic acids, including the intermediates of the tricarboxylic acid cycle; fatty acids; carbohydrates; and polyols) and some semi-polar secondary metabolites, such as phenylpropanoids, flavonoids, mono-, sesqui-, di- and even polycyclic triterpenes (sterols) and tocopherols [41,42,43]. As the representatives of plant metabolome often appears to be involved in adaptive metabolic adjustment in terms of the long-term plant stress adaptation [44], the comprehensive profiling of low molecular weight polar and semi-polar metabolites with GC-MS made it the method of choice in the biology of plant response to environmental stress, development, plant–animal and plant–microbial interactions [4,14,45,46]. 

It is worth noting that secondary compounds in contrast to the primary ones usually are much more diverse in plant organisms, although they are synthesized in much smaller quantities than compounds of primary metabolism. The diversity of their physicochemical properties, low concentrations and potential matrix interferences make analyzing many of secondary metabolites with conventional GC-MS a real challenge. The capabilities of one-dimensional GC-MS in secondary metabolome analysis can be significantly enhanced by the application of comprehensive two-dimensional gas chromatography combining with time-of-flight mass spectrometry (GC×GC-TOF-MS, 2D GC-MS). This technique is considered effective for analyzing complex mixtures of secondary metabolites, offering vastly increased separation (peak capacity) over traditional GC-MS by using two different columns (e.g., nonpolar/polar) for enhanced resolution especially compounds like terpenes and phenolics. Indeed, 2D GC-MS has exceptional informational power due to its enhanced analyte detectability. It allows us to detect and determine the content of a cluster of metabolites that share specific features [47,48]. In addition, the algorithms for the processing of GC×GC-MS data have recently been improved, also allowing for simplified metabolite identification [49]. Thus, Gastão-Muchecha and colleagues analyzed the volatile compounds in red wines by headspace solid-phase microextraction (HS-SPME) coupled with GC×GC-TOFMS and observed distinct terpene and C13-norisoprenoid profiles in the wine samples studied [48]. In another work, 2D GC-MS was employed for profiling essential oils from the naturally infected tropical tree *Aquilaria malaccensis* [50]. This experiment resulted in the detection of about 500 compounds, of which 150 were identified. Most of these metabolites were oxygenated and non-oxygenated mono- and sesquiterpenes, norterpenoids and diterpenoids. Generally, the valuable information obtained from 2D GC-MS experiments can be used to determine the main metabolic pathways and analyze the molecular mechanisms that link some secondary metabolites to the end plant phenotypes. 

However, despite the evident progress in optimizing 1D and 2D GC-MS methods and in establishing strategies for analyzing chromatograms, detecting analytes, and processing data, the future prospects of those methods lie in their combination with other approaches [51,52]. This is because the GC-MS-based metabolomics has some intrinsic limitations [53,54]. As it was mentioned above, several groups of primary and secondary metabolites appear to be challenging for GC-MS analysis because of their non-volatility, even in form of TMS-derivatives, due to their high molecular weight and/or thermal instability. Because of this, the metabolomics datasets acquired by GC-MS may not contain an essential portion of metabolites that could be crucial for the pathways underlying the phenomenon under study.

The latter aspect should not be underestimated. Indeed, for example, sugar phosphates (which are the intermediates of important central pathways—glycolysis, pentose phosphate and Calvin cycles), nucleotides and some cofactors do not form thermally stable derivatives under the conditions of standard sample preparation protocols and/or their derivatives do not sustain the high temperatures required for liquid GC injection in the split–splitless (SSL) injectors and further separation in temperature gradients [55,56]. The high complexity of the sugar derivatization patterns, which are represented with at least two reaction products per sugar metabolite, appears to be a less pronounced, although quite important problem [57,58]. In addition, analyzing glycosides (sugar-conjugated secondary metabolites) by GC-MS is challenging due to their thermal lability. Because of these reasons, to get the idea about the dynamics of the primary and secondary metabolisms (or at least to cover a major part of their principal pathways), the GC-MS analysis needs to be complemented with at least one other hyphenated technique, which provides direct access (i.e., without prior derivatization) to thermally labile metabolites. Indeed, HILIC-MS and IP-RP-HPLC-MS are often preferred for polar primary compounds, and RP-HPLC-MS for semi-polar secondary metabolites, as they are typically well retained on the reversed phase [52,59]. In the following chapter we will discuss these and other instrumental platforms that are often used as complementary techniques to GC-MS to improve metabolome coverage. 

### 2.4. Complementary Techniques to GC-MS

#### 2.4.1. HILIC-ESI-MS

One of the most suitable candidates for such a platform is hydrophilic interaction liquid chromatography, coupled online to electrospray ionization mass spectrometry (HILIC-ESI-MS) [60,61]. As this technique relies on the mobile phases based on organic solvents with high dipole moment (acetonitrile, methanol or isopropanol), it allows (with the implementation of appropriate desolvation techniques) the best possible ionization efficiency in the ESI source and, hence, the best accessible MS-sensitivity [62]. Over the last decade, it was successfully applied for comprehensive profiling of primary metabolites (especially amino acids), sugars, sugar acids and phosphates, lipids and many other metabolomics applications [63,64,65,66,67,68]. 

The information on successful integration of HILIC-MS and GC-MS datasets acquired with the same sample sets is limited and is available mostly in the clinical context. Thus, recently, Macioszek and colleagues reported a multiplatform study (comprising RP-LC-MS, HILIC-MS and GC-MS) to address the differences between individual KIT (receptor tyrosine kinase) mutants and assessing the impact of imatinib treatment on gastrointestinal stromal tumor tissues in mice [69]. The authors highlighted the stronger, in comparison to HILIC, potential of GC-MS for the analysis of polar metabolites, although the metabolome coverage of these two techniques was only partly complementary. Thus, acylcarnitines, characteristic for the tissue response to the imatinib treatment, were available only in the HILIC dataset. The same protocol was employed for comprehensive profiling of urine metabolome of cancer patients [70]. In that study, the complementary character of HILIC was more pronounced—this technique appeared to be the most suited for the analysis of the glucuronated metabolome. Importantly, HILIC was efficient in the analysis of complex conjugated metabolites with the molecular weights of up to 4000 Da, whereas GC-MS performed well with small molecules below 500 Da.

Recently, the multiplatform combination, including RP-LC-MS, HILIC-MS and GC-MS, was transferred to plants. Thus, Siddajah and colleagues applied the combination of these techniques to the comprehensive characterization of the *Alangium salviifoliums* bark metabolome [71]. The authors reported an annotation of 81 metabolites by their characteristic accurate *m*/*z* and fragmentation patterns. Obviously, implementation of the quantitative approach is required as the next step of the GC-MS/HILIC-MS-based plant integrative metabolomics.

Unfortunately, despite the great potential of HILIC for analysis of plant polar metabolites, it has some intrinsic limitations. First, being normal phase distributional chromatography, this method is featured with relatively slow kinetics of analyte interaction with stationary phase [72]. It results in long run times, high consumption of expensive organic solvents and compromised chromatographic efficiency that leads to unfavorably high peak width [73,74,75]. The latter fact negatively affects chromatographic resolution, metabolome coverage and sensitivity (due to reduced peak heights). Not less importantly, HILIC is associated with strong sample solubility problems and suffers from complex patterns of unspecific “non-HILIC” interactions of analytes with stationary phase, which affect its retention mechanism [76,77]. To overcome these excessive interactions, sophisticated buffer-based strategies need to be implemented [78]. Despite the recent introduction of UHPLC technology and improved amide-based phases in HILIC practice [79,80], the overall performance of the method is still below the standards universally accepted for RP-UHPLC [81]. Therefore, overcoming the major limitation of the reversed phase chromatography (RPC), poor retention of hydrophilic compounds, might be considered a promising strategy for analysis of polar thermolabile compounds, which can be treated as an elegant alternative to HILIC.

#### 2.4.2. (IP)-RP-(U)HPLC-MS

Retention of polar metabolites can be dramatically improved by application of ion-pair RPC (IP-RP-(U)HPLC) with alkylamines as ion pair agents [82]. In its most comprehensive version, this method was established in 2000s by the Rabinowitz group [83], and to date it is well established for the analysis of several key classes of polar primary compounds involved in central and energy metabolism, including nucleotides, coenzyme A esters, sugar nucleotides, amino acids, sugar phosphates and bisphosphates [84], with further extension to oligonucleotides [85]. This technique can be efficiently combined with the established GC-MS methodological platform [4,86]. 

One of the first such studies employing simultaneously these two techniques was reported at the beginning of the last decade by Dietl and colleagues [87]. The authors addressed the role of lactate in tumor necrosis factor (TNF) secretion and glycolysis flux. Therefore, the general metabolite profiling was done with GC-MS, whereas the lactate conversion was performed by IP-HPLC-QqQ-MS/MS.

Later, the results acquired by GC-quadrupole mass spectrometry with EI ionization (GC-EI-Q-MS) and IP-RP-HPLC-QqQ-MS/MS could be successfully integrated during the data processing step [88]. In terms of this approach, after the integration of the characteristic analyte peak areas in the corresponding extracted ion chromatograms (XICs), the result tables obtained with both methods were combined in one metabolite matrix prior to the post-processing step. Recently, Shumilina and colleagues demonstrated that GC-EI-Q-MS revealed 321 thermally stable primary metabolites in the aqueous methanolic extracts from cucumber roots, whereas the IP-RP-HPLC-QqQ-MS-based approach revealed 157 thermally labile metabolites. After merging the two corresponding datasets, a combined result matrix with 391 entries was built and processed with the MetaboAnalyst 5.0 online software tool. Thus, in this case, the integration of two metabolomics approaches occurs at the level of statistical and bioinformatics (functional) analysis [20]. Therefore, differentially abundant metabolites were identified, and the biological context of these alterations could be addressed by the pathway and enrichment analysis [20].

In addition, an integration of various (U)HPLC methods with 2D GC-MS appeared to be useful in multiple applications. Thus, a complex separation process using normal phase liquid chromatography coupled online with 2D GC-MS (LC-GC×GC) in combination with a dual detection system including single quadrupole (QMS) and flame ionization detection (FID) was applied for the qualitative and quantitative analysis of mineral oil hydrocarbons in cosmetics [89]. The combination of 2D GC-MS with high-resolution time-of-flight mass spectrometry (LC-GC×GC-HRTOF-MS) made it possible to determine the composition of minor components of animal fat [90]. 

#### 2.4.3. Capillary Electrophoresis (CE)-MS and Ion Chromatography (IC)-MS

Capillary electrophoresis–mass spectrometry (CE-MS) is another promising methodological platform, which can be used complementary to GC-MS for improving the quality and completeness of metabolomics datasets. Indeed, these two techniques appeared to have comparable performance in metabolite profiling [91]. Thus, in early studies, a high level of metabolome coverage similarity was found when CE-MS and GC-MS were used for the profiling of amino acids in suspension cultures of *Medicago truncatula* cells. In these early experiments, CE-MS proved to be an acceptable alternative to GC-MS for the targeted profiling of metabolites [35]. Later on, CE-MS was shown to be a powerful method for the separation of both cationic (amino acids, nucleosides and small peptides) and anionic (sugar phosphates, nucleotides and organic acids) metabolites [92]. As some of these metabolites (e.g., peptides and sugar phosphates) are hardly accessible by GC-MS, CE-MS can be considered as complementary to the GC-MS technique. 

Further, Kim and colleagues reported parallel GC-TOF-MS and CE-TOF-MS-based comprehensive metabolite profiling of the fast-fermented bean paste (cheonggukjang) inoculated with *Bacillus* strains to address the accompanying metabolic changes as a function of fermentation time [93]. The application of these two separation methods appeared to be really complementary: among the 123 metabolites discovered, 55% were assigned by GC-MS, and 45% were annotated by CE-MS. On the other hand, these two techniques can be complementary for the characterization of one specific metabolite group, as has been recently shown for extraction-free capillary electrophoresis coupled to mass spectrometry (CE-MS). This complementary character was also shown for direct immersion-solid-phase microextraction (DI-SPME) in combination with GC-MS (DI-SPME-GC-MS), as applied simultaneously to the analysis of biogenic amines in wines [94].

Another approach to address the contents of individual sugar phosphates in plant tissues is ion chromatography (IC)-MS. For example, glycolytic intermediates—pyruvate, glucose-6-phosphate, fructose-6-phosphate, fructose-1,6-bisphosphate, phosphoenolpyruvate, and the sum of 2-phosphoglycerate (2PG) and 3-phosphoglycerate (3PG)—can be reliably quantified by IC-tandem mass spectrometry (MS/MS). However, 2PG and 3PG (which co-elute under the IC conditions) can be efficiently separated and precisely analyzed by GC-MS. The same is the case for glyceraldehyde 3-phosphate (GAP) and diacylglycerol diphosphate (DGPP), which are likely to break down in the IC column due to the high concentration of sodium hydroxide used in the gradient [95]. 

#### 2.4.4. Cutting-Edge MS-Based Technologies

Despite the fact that modern approaches to metabolomics are quite informative, they still meet many challenges, the most important of which are coverage, the annotation of metabolites and their spatial distribution in plant tissues [1]. The development of novel MS-based technologies aims at overcoming these limitations. Thus, ion mobility spectrometry (IMS) is an analytical technique that may significantly expand the analytical potential of conventional GC-MS, 2D GC-MS or their combinations with LC-MS-based methods. IMS allows the separation of metabolites and their isomers based on their mass fragmentation structure and CCS (collision cross-section) values [96]. The method of direct combination of GC-IMS was successfully applied to identify volatile organic compounds (VOCs) of various natures—hydrocarbons, alcohols, ketones, aldehydes, esters and terpenes [97,98,99]. Using this method, key metabolites characteristic of Maillard reactions and lipid oxidation affecting taste sensations were identified [97,99]. The use of GC×GC-TOF-MS in combination with UPLC-IMS-QTOF-MS, employing additionally ion mobility mass spectrometry (IMS), allowed the distinction of the isomeric structures of co-released secondary metabolites and highlighted metabolites with different content levels in leaf and head types of lettuce, suggesting metabolic variations between the compared types of lettuce cultivars [49].

Spatial metabolomics based on MS imaging (MSI) is used to precisely localize metabolite distribution in plant tissues [1,100]. Combined with the information provided by the GC-MS approach, the use of spatial metabolomics makes it possible to map molecular mechanisms with cellular domains [101]. Thus, matrix-assisted laser desorption/ionization (MALDI)-MSI has established itself as a powerful method, in combination with the traditional GC-MS analysis of the metabolism of the entire tissue, to obtain a detailed understanding of the plant response to water-deficit stress [101]. Understanding spatiotemporal differences in metabolic responses to stress can be used to engineer targeted strategies for developing plants with desired properties. However, to understand the synthesis, accumulation, and cross-regulation of metabolites in plants, it is necessary to visualize the distribution of metabolites in plant tissues with high spatial resolution [102]. 

### 2.5. Spectroscopic Techniques

Despite the fact that the combination of GC-MS with other MS-based techniques is obviously advantageous in terms of metabolome coverage, such setups still suffer from limited structural information, i.e., multiple analytes remain annotated as unknowns. Fortunately, this situation can be essentially improved by implementation of spectroscopic techniques in the MS-based multiplatform workflows [103]. 

For example, an integrated GC-MS- and LC-MS-based approach can be efficiently combined with spectroscopic techniques for analysis of polysaccharides [104,105,106]. Expectedly, this strategy could give access to high-quality, detailed structural information. For example, Honda and colleagues employed it for the analysis of acid hydrolysates prepared with extracellular polysaccharide (ECP) after its isolation from tuberose (*Polianthes tuberosa*) callus cultures [106]. These analyses employed ion exchange column chromatography with sub-sequent GC-MS analysis of the unretained component and two acidic hydrolysate fractions (TPS-1 and 2) to access the contents of its constituent monosaccharides—arabinose, mannose, galactose and glucuronic acid. This dataset was complemented with ^1^H and ^13^C NMR spectroscopy after the methylation of the polymeric sample [106].

The advantages of NMR spectroscopy as the tool for structural characterization of natural products are universally recognized: currently, it is the method of choice and the gold standard for the most reliable feature identification [39,107,108]. Unfortunately, this method is limited to the detection of the most abundant metabolites, whereas MS allows the discovery, tentative annotation and quantification of natural products at very low concentrations. One needs to take into account, however, that MS provides only structure confirmation but not the unambiguous identification of plant metabolites. This makes NMR spectroscopy and MS highly complementary tools in state-of-the-art metabolomics. It is therefore clear, that the combination of these two methodological platforms is likely to improve the overall quality of the study and expand the scope of metabolome applications [103,109]. Thus, due to the obvious advantages of NMR—high productivity, minimal sample preparation required, reasonable time for analyzing a higher amount of samples—it can be used as a promising extension of the regular GC-MS-based metabolomics workflow to gain a deeper insight in the molecular mechanisms underlying changes in the metabolome [103,110,111]. 

In particular, Barding and colleagues addressed the stress-associated metabolism rearrangements using ^1^H NMR and GC-MS to understand the complex biochemical and molecular response of rice plants to water immersion [111]. The high dynamic range of NMR, as compared with that of the GC-TOF-MS, provided a broad coverage of the metabolome within a single experiment. Some metabolites, such as S-methylmethionine and the dipeptide alanylglycine, could be detected and quantified only by ^1^H NMR. The sensitivity of GC-MS allowed the quantification of sugars, organic acids and amino acids, some of which were not detected by NMR, and provided additional information on the regulation of the TCA cycle [111].

As a metabolite profiling tool, NMR is featured with high accuracy and reproducibility, whereas the limited sensitivity of NMR can be efficiently compensated by the use of multivariate statistics [112]. Moreover, vendor-specific software packages for interpretation of the NMR data are available. 

To summarize, integrated analytical techniques essentially enlarge the spectrum of detected and efficiently quantified metabolites in comparison to the metabolomics studies based on a single analytical method [113]. Thus, most often, the different metabolomic approaches complement each other, and the combination of GC-MS with other metabolomic approaches is a promising and important strategy for metabolomic research aimed at the widest possible coverage of plants’ metabolome. To integrate the information from several datasets acquired by different analytical methods, bioinformatics approaches and data analysis tools are required [114]. Despite the impressive development of instrumental setups and data handling strategies, this task still appears to be challenging to date and will be discussed in more detail in the following chapter. However, it should be pointed out that there have been some software packages developed so far, such as Agilent Mass Profiler Professional (MPP, Agilent Technology [115]), which has a variety of useful visualization and quantification tools for analyzing metabolomics data. Importantly, this software gives access to the parallel analysis of metabolomics data acquired by LC-MS, GC-MS, CE-MS, ICP-MS and NMR within one project. 

## 3. Implementation of GC-MS in Multi-Omics Strategies of Post-Genomic Analysis

### 3.1. Metabolomics-Centered Position in Multi-Omics Integration

It is well-known that all methodological platforms of post-genomic research (often referred to as omics technologies) represent the valuable sources of rich functional information, which provide insights deep inside into the dynamics of transcripts, proteins and metabolites in the living cell. However, besides the obvious advantages, all these platforms have intrinsic limitations, mostly associated with sample preparation, sensitivity, precision and reproducibility issues. The combination of several post-genomic platforms in one workflow allows the mutual cross-complementation of these limitations. It makes the integrated workflow more powerful and the whole data processing strategy more robust and less prone to the generation of false results due to the possibility for cross-validation of the corresponding datasets [39]. On the other hand, the possibility of inter-omics data cross-validation within the multi-omics setups essentially improves the reliability of each individual complementing omics technique.

In the context of the data reliability and quality gain, the inter-omics cross-validation appears to be especially useful for metabolomics. Indeed, among the all-global molecular pools—transcriptome, proteome and metabolome—the latter is featured with the highest plasticity, which is mostly underlined by fast enzyme kinetics and, hence, high rates of metabolite conversion. Because of this reason, plant metabolome is actually a unique snapshot of the intermediates, generated by a complex dynamic metabolite network at the specified moment. Therefore, plant metabolome, assessed in two different (even very close) time points, might be rather different. Due to this highly dynamic character of the metabolome, very often metabolomics data are insufficient to understand the long-term regulatory mechanisms involved in regulation of the metabolic pathways of interest [116]. Therefore, to get a deeper insight into the plant metabolome and to address the physiological role of specific metabolic pathways and/or individual metabolites in a more comprehensive way, metabolomics data need to be considered together with the outputs of transcriptomics and proteomics experiments.

In terms of such multi-omics strategies, metabolomics plays the central role in the characterization of the current physiological and metabolic status of the plant, as well as its direct role in plant phenotype, whereas the other omics techniques have more impact on the establishment of the molecular mechanisms behind the observed metabolome dynamics, especially in the context of the information encoded in genome [6]. Thus, within the multi-omics workflows, metabolomics occupies the most central “heart” position of the whole pipeline, as it gives the critical information about the affected metabolic pathways. At the further step, proteomics and transcriptomics might deliver not only important mechanistic information but also the response-specific markers of the accompanying alterations in the cellular metabolism. As these markers are expected to be more reproducible in time than metabolites, they can contribute to an elucidation of the associations between the omics variables and underlying biological functions. This would finally help to achieve another goal of the multi-omics approach: to provide an essential readout of biological processes for metabolism reprogramming. 

### 3.2. Applications and Impact of GC-MS in Multi-Omics Plant Research

The availability and rapid development of high throughput and cost-efficient platforms made generation of massive multi-omics data possible. Due to this, the multi-omics strategy has gained growing interest among plant scientists over the recent decade, resulting in an increasing number of reported integrated multi-omics datasets. GC-MS, as one of the powerful analytical techniques in the metabolomics toolbox, was successively applied alone [12,13] or in combination with other metabolomics platforms (e.g., UHPLC-MS [6], NMR [110] and CE-MS [95]) in integrative multi-omics analysis (Figure 1). It contributes to the connection of individual omics variables (metabolites, proteins, transcripts, genes) in a network to predict phenotypic traits [117] or the alteration of a phenotype associated with disease [13] or environmental stress [118]. In the establishment of such a connection, GC-MS plays an important and well-defined role. Indeed, as this technique covers most of the primary and some groups of secondary metabolites of energetic and central biosynthetic pathways, it became widely implemented in multiple large-scale multi-omics studies aiming at understanding development-dependent reprogramming, in particular seedling growth and its modulation by growth-promoting agents [119], seed maturation and storage [59], post-harvest fruit physiology [12] and stress tolerance mechanisms in different plant systems [120]. 

Another rationale for the implementation of GC-MS in multi-omics research is its valuable impact on crop breeding programs. In this aspect, metabolites are considered as integrators in the complex interaction between the plant genotype and the environment. The interpretation of the metabolomics data collected from plants grown in different environments might be helpful in the prediction of the valuable from the agronomic point of view phenotypes and in the identification of the metabolites that can be used as biomarkers for these traits. This approach, combined with related genomics information, allows the accurate identification of the genes and metabolic pathways involved in the formation of agronomically important traits that can be applied in crop breeding programs [116,117].

### 3.3. Challenges of Multi-Omics Data Integration

However, despite the growing interest to the multi-omics approach in scientific communities, integration of several datasets acquired by multiple omics platforms still remains a challenging task [7]. Indeed, on one hand, the inter-omics comparisons of experimental designs, instrumental platforms and analytical procedures are not straightforward. Therefore, direct comparisons are difficult, and proper integration upon such direct comparisons is hardly possible without the proper normalization of all steps of the designed experiment (sample preparation, instrumental analysis, data mining) that preceded the multi-source data integration. The issues to be considered include limited amounts of tissue samples; high heterogeneity of biological material; differences in abundances and analytical responses of specific biomolecules in the samples; differences in the sample storage regimen and processing; technical artifacts, including batch effects and background contamination; and differences in the format of integrating omics datasets resulted from measurements of fundamentally different biomolecules. 

To overcome at least some of these issues, a list of minimal requirements to ensure dataset compatibility was developed by the society [5]. Thus, the proposed guidelines relied on the experimental design and analytical workflow specific for metabolomics, which is not surprising in the context of its above-mentioned central position in multi-omics workflows due to the largest contribution of metabolite patterns in phenotype. No less importantly, the metabolomics experimental design appeared to be the most compatible with the other post-genomic platforms. Therefore, following this recommendation would significantly improve the output of multi-omics research. 

Another important requirement to be followed to secure the high-quality of multi-omics data integration is inter-set consistence at the level of the plant material. Thus, all parallel omics experiments need to be accomplished with the same plant material, i.e., all analyses need to rely on the same sample set. Ideally, the frozen milled material is weighed from the same tube in several method-specific aliquots.

A comprehensive literature mining and the development of a well-formulated initial central hypothesis to be addressed in the whole experiment also essentially contributes to the success of a large-scale multi-omics experiment. At this step, a decision about the desired strategy (i.e., experiment type) for integrating the datasets from multiple omics platforms needs to be taken. 

**Figure 2:**
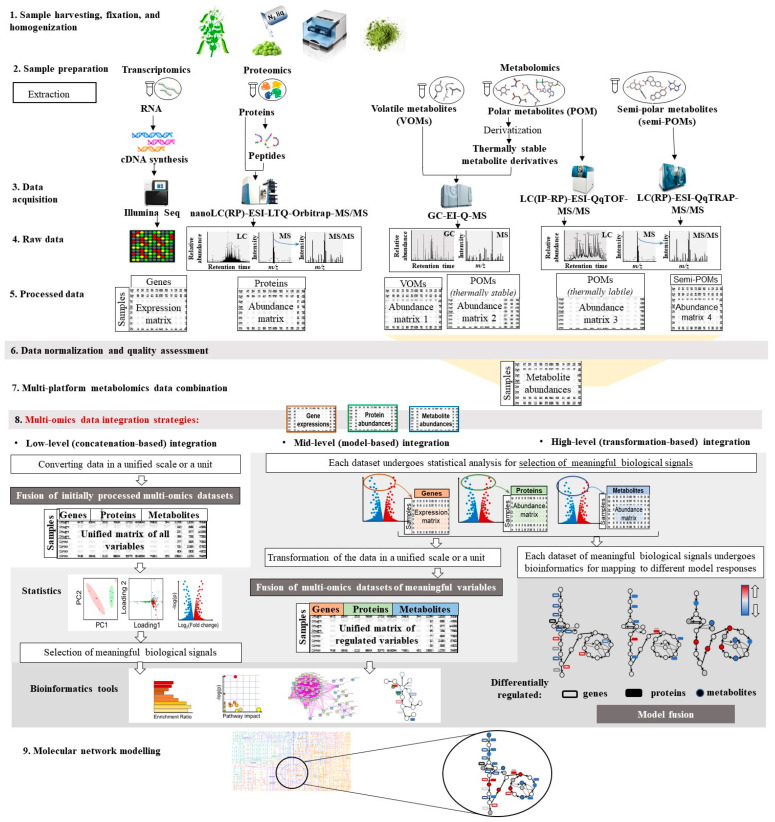
General workflow of plant multi-omics experiment (steps 1–9) and types of data integration strategies (step 8) employed in multi-omics experiments, including those implementing GC-MS-based metabolomics. The experiment workflow includes the following steps: (1) sample harvesting, fixation, and homogenization; (2) sample preparation; (3) data acquisition; (4) obtaining raw data of various formats; (5) data processing; (6) data normalization and quality assessment; (7) multi-platform metabolomics data combination; (8) multi-omics data integration data; (9) molecular network modelling. Multi-omics integration strategies: low-level integration (also called concatenation-based integration) depicts concatenation of individual omics datasets in a joined matrix for construction of an integrated model (left); mid-level integration assumes first mapping of individual omics sets or their transformation to obtain the data matrixes of less dimensionality, which serve as the input for further construction of an integrated model (middle); and high-level integration assumes generation of separate models from each dataset (right). The models are used at the next step to construct an integrated model. Abbreviations for metabolomics analytical platforms: nanoLC(RP) –ESI-LTQ-orbitrap-MS/MS–nano-flow liquid chromatography(reversed phase)–electrospray ionization-linear ion trap-orbitrap tandem mass spectrometry; nanoLC(IP-RP)–ESI-QqTOF-MS/MS–nano-flow liquid chromatography(ion-pared-reversed phase)–electrospray ionization-quadrupole- time-of-flight tandem mass spectrometry; nanoLC(RP)–ESI-QqTRAP-MS/MS–nano-flow liquid chromatography(reversed phase)-electrospray ionization–quadrupole-ion trap tandem mass spectrometry; GC-EI-Q-MS–gas chromatography–electron ionization-quadrupole mass spectrometry.

In general, the plant multi-omics experiment workflow involves certain steps, each of which is critical to the success of the study (Figure 2). The various challenges associated with integrating multi-omics data often relate to incomplete data characteristics, low correlation between data measured on separate platforms and various approaches to normalizing and transforming data for different omics. Therefore, the omics-specific standardized preprocessing and normalization of obtained data is also a key aspect of successful integration of multi-omics data. This takes into account differences in measurement methods such as systematic variations in sample preparations, sample volumes and instrument responses, while preserving the true biological variability between sample groups. For example, quantile normalization is used for transcriptomics; median centering for proteomics; and probability coefficient normalization, internal and external standard normalization and sample-based (fresh weight, dry weight) normalization are of often used in metabolomics research [121]. 

Furthermore, before the integration of data originated from different analytical instruments, quality assessment across those datasets is necessary to detect systematic instrument-specific technical biases, potential batch effects and outliers, since they will mask underlying biological signals in each dataset that may result in misleading of data interpretation. The reliability of obtained data is assessed via the use of quality control samples (QC) that should run alongside experiments and therefore be incorporated in design of each analysis. Importantly, the QC should satisfy the omics-specific requirements [122]. Generally, a few specialized types of QC have been developed for evaluating different aspects of instrument performance (system suitability, process monitoring and long-term stability) and data reproducibility [123]. Thus, internal QC samples present “spike-in” standards that spike into experimental samples, they help assessing deviations at abundance levels of metabolites, peptides, proteins or RNA molecules introduced during sample preparation and also help in evaluating instrument functionality [122]. External QC samples, which are typically pooled biological samples, are used for monitoring instrumental performance shifts, detecting batch effects and improving data quality [124]. They are inserted periodically within the same batch during acquisition to track stability and variations over time, ensuring data reliability by highlighting issues from sample preparation and acquisition. 

Finally, integrating multi-omics data may be a challenge because of other critical technical issues such as cross-platform data normalization and quantitative consistency. Most of the issues are due to heterogeneity of data originated from different instruments. Indeed, different platforms produce primary output metrics with inherently different structures and distributions. Thus, RNA-seq yields count values, microarrays produce fluorescence intensities, while MS-based metabolomics and proteomics quantify molecules as relative or absolute abundance values. These distinct data types reflect their underlying mechanisms—sequencing reads, hybridization signals and mass-to-charge (*m*/*z*) detection—leading to different data characteristics, such as dynamic range and sensitivity. Nevertheless, they often show strong correlation in expression levels. Modern bioinformatics tools aim to integrate these diverse datasets using machine learning algorithms to obtain a more complete biological picture: mixOmics [125], MetaboAnalyst [126], INTEGRATE [127] and Onassis [128]. For that, the primary data have to be first standardized and harmonized to ensure their compatibility [129]. These steps, besides data normalization and the data quality assessment, which were already mentioned above, also include converting or mapping data to a common scale or units or references [114,130]. However, there are integration strategies that are not only based on the direct integration of the processed multi-omics data; they will be discussed in the following chapters. 

### 3.4. Selection of a Multi-Omics Data Integration Strategy: An Overview of Approaches

#### 3.4.1. Multi-Level Data Integration

To date, several classifications of multi-omics data integration types are known. One of them categorize the integration analyses with respect of the type of the input data, subjected for integration (Figure 2): low-, mid- and high-level data integration (or data fusion) [6]. When the low-level integration (also known as concatenation-based integration) is employed, row or processed data obtained by different omics technologies are combined in a total matrix for further statistical analysis and/or molecular network modeling. This approach gives access to the most complete use of the acquired information and allows preserving information about the interaction between variables from different omics platforms. Thus, finding the way for an appropriate common representation for datasets from different scales represents the main challenge of this type of the inter-omics data integration. The issue was successfully overcome by López-Hidalgo and colleagues, who applied this type of integration to visualize and partially reconstruct the metabolism in holm oak [114]. In that study, data, i.e., omics variables—transcripts, proteins and metabolites—obtained from analyses of transcriptome (by Next-Generation Sequencing, NGS-Illumina), proteome (by shotgun nano-flow liquid chromatography–tandem mass spectrometry, nanoLC-MS/MS) and metabolome (by GC-MS) were integrated by their Enzyme Commission numbers (EC numbers for transcripts and proteins) and Kyoto Encyclopedia of Genes and Genomes (KEGG) identifiers (GC-MS metabolites) to further obtain a holm oak metabolic overview using the bioinformatic tool MapMan. 

The mid-level data fusion (also known as the model-based integration) assumes combination of the datasets generated by different omics sources at the data level after selection of meaningful variables [6]. In terms of the concept of high-level data integration (also known as transformation-based integration), original omics datasets are first transformed or mapped to different model responses to indicate relationships between the treatment groups. Afterwards, the models are fused to produce a final response integrative model [6]. For mapping and integration steps, multiple graph- (or network-) based and kernel-based bioinformation algorithms are used [130]. The graph-based methods represent relationships between the sample groups by denoting subjects by nodes and relationships between them by edges. This type of representation provides an easy and straightforward way of data integration. This approach provides good data interpretability and becomes, therefore, widely used in omics data integration. Kernel-based methods are used for analysis of high-dimensional data. These methods consider nonlinear effects, and were successfully applied to the capture of important data type-specific effects [130]. The kernel-based approaches have strong prediction power [131].

The implementation of the GC-MS-based workflow in the mid-level and high-level integration strategies, supported by the graph-based methods, can be exemplified by numerous studies [12,13,132]. Thus, in a study addressing the effect of the exogenous methyl jasmonate application on the fresh tea leaves, a combination of GC-MS and UHPLC-MS in parallel to isobaric tags for relative and absolute quantification (iTRAQ)-based proteomics analysis was employed [132]. The subsequent statistical interpretation of the acquired metabolomics and proteomics datasets allowed detection of 100 volatile and 266 nonvolatile metabolites as differentially abundant in addition to a rich pattern of differentially expressed proteins. These differentially abundant outputs of both proteomics and metabolomics were integrated into one resulting set by KEGG mapping. Based on the raw KEGG maps, the authors designed self-constructed maps, which provided explicit visualization of key changes in major biosynthetic pathways of volatile compounds associated with tea aroma quality. 

The strategy of high-level integration of two omics datasets (GC-MS-based metabolomics and iTRAQ/nano-flow liquid chromatography–mass spectrometry (nanoLC-MS)-based proteomics) was successfully employed by Yang and colleagues for the elucidation of the metabolite and protein dynamics in the rat hippocampus after chronic social defeat stress [13]. The original GC-MS-based metabolomics dataset was first analyzed by PCA and orthogonal partial least squares discriminant analysis (OPLS-DA) to identify the differentially abundant metabolites. At the next step, pathway and enrichment analyses were performed for these regulated metabolites. These analyses yielded preliminary information on the rat metabolic pathways, which were affected by stress treatment. Analogously, all proteins detected in the study were filtered to select a set of differentially expressed polypeptides, which were subjected to hierarchical clustering, gene ontology (GO), KEGG enrichment and protein–protein interaction (STRING) analyses. These efforts resulted in identification of the pathways, which were putatively associated with depression rat phenotype. At the final step, an ingenuity pathway analysis (including canonical pathway and molecular interaction network analyses) of integrated metabolites and proteins was applied. As the result, intracellular signal transduction cascades and related secondary messengers could be highlighted as the most significantly altered in the hippocampus of the depressed rats [13]. One needs to take into account, however, that the high-level integration strategy has some limitations. Thus, as initial models are designed independently, some important information about the potential inter-omics interactions can be lost.

#### 3.4.2. Multi-Omics Data Integration: From Understanding the Phenotype to Predicting Biological Mechanisms

Another classification of the integration strategies might rely on the focus of the multi-omics study. In this sense, the multi-omics data integration strategies can be categorized as sequential integration, biological analysis and model-based analysis [6]. 

The first strategy aims at finding the reason for the development of a certain phenotype rather than the prediction of its appearance. The key questions addressed by this strategy are: (i) how does the multi-omics data integration help to enhance understanding of the phenotype and (ii) would the application of additional omics data validate the results obtained from each individual omics dataset? The sequential integration typically follows the following conventional scheme: the first step (i) assumes recognition of the genes associated with the phenotypic response to environmental or exogenous treatments/stimuli and integration of the related omics data, (ii) then the genes that can be attributed to specific metabolites and proteins are incorporated into the integration pipeline. The final step (iii) involves integrating the genes, proteins and metabolites that were previously annotated to corresponding metabolic pathways highlighted by enrichment analysis as pathways of interest, which are most strongly involved in the target response. This strategy is also well applicable to the discovery of biomarkers characteristic of specific phenotype traits. The sequential integration was applied in a number of studies, including those employing the GC-MS-based metabolomics setup [111,112]. 

The model-based integration approach relies on the statistics and machine learning methods to prove capacity of the integrative model. This strategy allows significant improvement of the prediction quality and is more resistant to technical and methodological biases. In this approach, methods of dimensionality reduction (canonical correlation analysis (CCA), principal component analysis (PCA) and partial least squares (PLS)-derived methods) are widely used, as they focus on finding correlation patterns (combinations between variables) and provide information in terms of common and orthogonal components [133]. However, it is not straightforward to quantify the associations between various types of variables, and therefore this limits the overall model-based integration with regard to explanations of mutual interrelationships between variables [6]. In biological integration, the interpretation of the results obtained by dimensionality reduction methods can be made easier by prior knowledge of the metabolism of the biological system under investigation. The main purpose of this approach is to establish molecular mechanisms (i.e., biological meaning) on interacting variables obtained from various omics platforms. Thus, biological integration focuses on uncovering the biological meanings (metabolic, regulatory, and signaling mechanisms) from the functional information acquired by interacting different omics variables for biological integration. In general, both strategies (model-based and biological integrations) can exploit the same panel of statistical methods for data integration. However, the former approach via the methods only seeks statistically significant associations, while the latter uses the methods in combination with biological knowledge to drive underlying biological mechanisms. 

The study by Acharjee and co-authors demonstrated the effectiveness of random forest regression in integrating multiple gene expression, LC-MS, GC-MS and proteomics data for the prediction of four quality traits of potato [117]. In terms of the biological integration setup, a special emphasis is put on the importance of the previously collected knowledge about the metabolism of the biological object in study. Thus, the previously collected information serves to enhance the interpretability of the resulting statistical output of interacting omics variables. Multiple multi-omics studies, including the GC-MS-based setups for the analysis of primary and secondary metabolites within a multi-omics experimental design, often employ the biological integration strategy. The reason for this might be the high analytical power of the GC-MS-based profiling of various metabolites. It allows relatively easy and straightforward mapping of the processes, potentially essential for maintaining cellular homeostasis, stress-induced adaptations, plant growth and development. Therefore, as GC-MS delivers this critical core information, in the integration workflows of this type, the corresponding datasets usually act as the starting point of omics data co-interpretation. Complementary information, delivered by other omics, allows for better understanding of the biological roles of the assigned pathways in terms of the associations found in multi-omics experiments.

## 4. Conclusions

Taken together, the use of multiple omics techniques for the generation and interpretation of the complementary datasets from the same samples provides a deeper insight in the molecular mechanisms behind the morphological and physiological responses, observable at the level of phenotype. Thus, the implementation of the GC-MS-based profiling of nonpolar, semi-polar and polar metabolites in the multi-omics workflows provides the key meaningful information. On the other hand, the multi-omics experiment brings multiple challenges regarding analytical procedures, data processing and the integration of the GC-MS data with other platforms. However, due to the development of new powerful analytical platforms, data science and bioinformatics, these challenges can be overcome. For this, special requirements for the proper design of multi-omics experiments were established in the post-genomics society. Nevertheless, currently, the multi-source data integration remains a real “bottleneck” in all multi-omics studies. Obviously, this aspect will be the mainstream of the multi-omics research of the coming decade. A perspective direction for developing a multi-omics approach using GC-MS may be the development of new algorithms that via integrated mapping can contribute to better annotation of unknown metabolites. Future progress in this area is likely linked not only to the development of specialized software tools, platforms for molecular networks and databases, but also to the application of artificial intelligence (AI) algorithms. It is possible that novel AI algorithms could facilitate the integration of multi-omics data for functional prediction purposes, such as the prediction of gene clusters responsible for certain metabolites.

Thus, new and more powerful algorithms need to be proposed for multi-omics data integration and interpretation. However, their diversity without comprehensive comparison of their performance makes the selection of appropriate tools still difficult.

## Data Availability

No new data were created or analyzed in this study.

## Abbreviations

2PG	2-phosphoglycerate
3PG	3-phosphoglycerate
CE	Capillary electrophoresis
CE-MS	Capillary electrophoresis–mass spectrometry
CE-TOF-MS	Capillary electrophoresis–time-of-flight-mass spectrometer
CoA	Coenzyme A
DGPP	Diacylglycerol diphosphate
DI-SPME-GC-MS	Direct immersion-solid-phase microextraction–gas chromatography–mass spectrometry
EC numbers	Enzyme Commission number
ECP	Extracellular polysaccharide
EI	Electron impact
ESI	Electrospray
GAP	Glyceraldehyde 3-phosphate
GC-EI-Q-MS	GC-quadrupole mass spectrometry with EI ionization
GC-FID	Gas chromatography–flame ionization detector
GC-MS	Gas chromatography–mass spectrometry
GC-TOF-MS	Gas chromatography combined with time-of-flight mass spectrometry
GO	Gene ontology
HS-SPME	Headspace solid-phase microextraction
HILIC	Hydrophilic interaction chromatography
HILIC-(U)HPLC	Hydrophilic interaction-high performance liquid chromatography
HILIC-ESI-MS	Hydrophilic interaction chromatography–electrospray-mass spectrometry
HILIC-MS	Hydrophilic interaction chromatography–mass spectrometry
IC	Ion chromatography
IC-MS/MS	Ion chromatography–tandem mass spectrometry
IP	Ion-paired
IP-HPLC-QqQ-MS/MS	Ion-paired high-performance liquid chromatography coupled with triple quadrupole tandem mass spectrometry
IP-RP-(U)HPLC	Ion-pair reversed-phase (ultra) high-performance liquid chromatography
iTRAQ	Isobaric tags for relative and absolute quantification
KEGG	Kyoto Encyclopedia of Genes and Genomes
KIT	Tyrosine kinase
LC-MS	Liquid chromatography–mass spectrometry
LC-Q-MS	Liquid chromatography–quadrupole mass spectrometry
LC-QqQ-MS	Liquid chromatography–triple quadrupole-mass spectrometry
LC-QqTOF	Liquid chromatography–quadrupole time-of-flight mass spectrometer
LC-QqTOF-MS	Liquid chromatography–quadrupole time-of-flight mass spectrometry
LC-QTRAP MALDI	Liquid chromatography–quadrupole ion trap mass spectrometer Matrix-assisted laser desorption/ionization
MPP	Mass Profiler Professional
mRNA	Messenger RNA
MS	Mass spectrometry
MSI	Mass spectrometry imaging
nanoLC-MS	nano-flow liquid chromatography–mass spectrometry
NGS	Next generation sequencing
NMR	Nuclear magnetic resonance
OPLS-DA	Orthogonal partial least squares-discriminant analysis
RP	Reversed-phase
RPC	Reversed-phase chromatography
RP-HPLC-MS	Reversed-phase high performance liquid chromatography–mass spectrometry
RP-LC-MS	Reversed-phase liquid chromatography–mass spectrometry
RP-UHPLC	Reversed-phase ultra-high-performance liquid chromatography
TCA cycle	Tricarboxylic acid cycle
TMS	Trimethylsilyl
TNF	Tumor necrosis factor
UHPLC	Ultra-high-performance liquid chromatography
UV-VIS	Ultraviolet–visible spectroscopy
XICs	Extracted ion chromatograms
